# Capsaicin induces cell cycle arrest and apoptosis in human KB cancer cells

**DOI:** 10.1186/1472-6882-13-46

**Published:** 2013-02-25

**Authors:** Chia-Han Lin, Wei-Cheng Lu, Che-Wei Wang, Ya-Chi Chan, Mu-Kuan Chen

**Affiliations:** 1 Department of Otorhinolaryngology, Head and Neck Surgery, Changhua Christian Hospital, 135 Nanhsiao St, Changhua, Taiwan; 2 Cancer Research Center, Changhua Christian Hospital, Changhua, Taiwan

**Keywords:** Capsaicin, KB cells, Cell cycle, Apoptosis, Mitochondria

## Abstract

**Background:**

Capsaicin, a pungent phytochemical in a variety of red peppers of the genus *Capsicum*, has shown an anti-proliferative effect on various human cancer cell lines. In contrast, capsaicin has also been considered to promote the growth of cancer cells. Thus, the effects of capsaicin on various cell types need to be explored. The anti-proliferative effects of capsaicin on human KB cancer cells are still unknown. Therefore, we examined the viability, cell cycle progression, and factors associated with apoptosis in KB cells treated with capsaicin.

**Methods:**

The cell proliferation/viability and cytotoxicity of KB cells exposed to capsaicin were determined by a sulforhodamine B colorimetric assay and trypan blue exclusion. Apoptosis was detected by Hoechst staining and confirmed by western blot analysis of poly-(ADP-ribose) polymerase cleavage. Cell cycle distribution and changes of the mitochondrial membrane potential were analyzed by flow cytometry. Furthermore, the expression of caspase 3, 8 and 9 was evaluated by immunoblotting.

**Results:**

We found that treatment of KB cells with capsaicin significantly reduced cell proliferation/viability and induced cell death in a dose-dependent manner compared with that in the untreated control. Cell cycle analysis indicated that exposure of KB cells to capsaicin resulted in cell cycle arrest at G_2_/M phase. Capsaicin-induced growth inhibition of KB cells appeared to be associated with induction of apoptosis. Moreover, capsaicin induced disruption of the mitochondrial membrane potential as well as activation of caspase 9, 3 and poly-(ADP-ribose) polymerase in KB cells.

**Conclusions:**

Our data demonstrate that capsaicin modulates cell cycle progression and induces apoptosis in human KB cancer cells through mitochondrial membrane permeabilization and caspase activation. These observations suggest an anti-cancer activity of capsaicin.

## Background

It is well known that natural phytochemicals widely present in certain daily consumed fruits and vegetables have inhibitory effects on various types of cancers at molecular and cellular levels [1,2]. Capsaicin, one of these naturally occurring phytochemicals, is the major pungent constituent of hot chili peppers of the genus *Capsicum* (family *Solanaceae*), which are extensively used as a food additive. It has been shown that capsaicin is involved in several physiological and pharmacological effects [3,4]. For example, several reports show that the use of capsaicin can relieve inflammation and pain associated with some diseases and cancer [5-7]. In addition, accumulating studies have shown that capsaicin has anti-proliferative effects on various human cancer cell lines including those derived from leukemia [8-10], multiple myeloma [11], cutaneous cell carcinoma [12], glioma [13,14], tongue cancer [15], nasopharyngeal carcinoma [16], esophageal carcinoma [17], gastric cancer [18], pancreatic cancer [19,20], hepatocarcinoma [21,22], colon carcinoma [23,24], non-small cell lung cancer [25], breast cancer [26,27], and prostate cancer [28,29]. The capacity of capsaicin to suppress the growth of these cancer cells is primarily mediated through induction of apoptosis. Additionally, the activities associated with capsaicin-induced anti-cancer effects include the arrest of cell cycle progression, regulation of transcription factor expression, and suppression of growth signal transduction pathways.

The failure to control cancer cell death associated with the induction of apoptosis has been considered to be a critical cause of resistance against cancer therapy [30]. Apoptosis, a type of programmed cell death, is a physiological homeostatic mechanism. As a result of apoptosis, unwanted cells are eliminated in a well-organized sequential process. Apoptosis is characterized by various morphological and biochemical changes such as pyknosis, plasma membrane blebbing, mitochondrial membrane permeability, and the activation of caspase cascades [31]. It has been shown that the activation of apoptosis is mainly mediated through the extrinsic death receptor pathway and the intrinsic mitochondrial pathway, which involve a variety of caspase family members [30-32]. The extrinsic pathway is initiated by stimulation of death receptors that are members of the tumor necrosis factor receptor family. Activated death receptors induce formation of the death-inducing signaling complex (DISC) that subsequently promotes activation of caspase-8. The intrinsic pathway initiated by various intracellular signals, such as DNA damage, involves the mitochondrial response. Disruption of the mitochondrial membrane through the regulation of the Bcl-2 family members dissipates the mitochondrial transmembrane potential, resulting in the release of proapoptotic proteins, including cytochrome c and apoptosis-inducing factor, from the intermembrane space into the cytosol. Consequently, the apoptosome, a complex that stems from the interaction between cytochrome c, apoptosis protease-activating factor 1 and ATP/dATP, activates caspase-9. Both extrinsic and intrinsic pathways induce the activation of caspase 3, 6 and 7 that subsequently cleave their substrates including poly-(ADP-ribose) polymerase (PARP), ultimately leading to apoptosis.

Despite our increasing understanding of the anti-cancer effects of capsaicin on the above-mentioned cancer cell lines, capsaicin has also been found to promote the growth of cancer cells [33,34]. The effects of capsaicin on various types of cancer need to be identified. The effect of capsaicin on human KB cancer cells remains unknown. Therefore, to gain insight into its effects, we determined whether exposure to capsaicin leads to cell cycle arrest and induction of apoptosis, and whether mitochondria and caspase members are involved in the programmed cell death. Here, we show that capsaicin induces arrest of the cell cycle at G_2_/M phase and causes apoptosis of KB cells. The capsaicin-induced apoptosis in KB cells is associated with mitochondrial membrane permeabilization and caspase activation. These results reveal that capsaicin may be useful for the prevention of cancer cell growth.

## Methods

### Cell culture and chemicals

Human KB cancer cells from the American Type Culture Collection (Manassas, VA, USA) were cultured in Dulbecco’s modified Eagle’s medium (GIBCO, Carlsbad, CA, USA) containing 10% fetal bovine serum, 100 U/ml penicillin, and 100 μg/ml streptomycin (GIBCO) at 37°C in a humidified atmosphere with 5% CO_2_. Capsaicin (Sigma, St. Louis, MO, USA) was dissolved in pure dimethyl sulfoxide (DMSO) (Sigma). All chemicals were of the highest grade available.

### Cell viability and cytotoxicity assays

KB cells (8×10^3^ cells/well) were seeded in 96-well plates and cultured overnight. The cells were treated with various concentrations of capsaicin (1, 50, 100, 150, 200 and 250 μM) or DMSO (control group). After 24, 48 and 72 h, cell proliferation and viability was determined by a sulforhodamine B (SRB) colorimetric assay [33]. Briefly, the cells were fixed in 10% w/v trichloroacetic acid (Sigma) and stained with 0.4% SRB (Sigma). The cells were then washed with tap water and 1% acetic acid (Merck, Darmstadt, Germany). Protein-bound precipitates were dissolved in 10 mM Tris buffer (pH 10.5) (Merck), and the plate was read at a wavelength of 492 nm (Multiskan Spectrum Microplate Reader; Thermo Labsystems, Waltham, MA, USA) to determine the cell viability. Trypan blue exclusion was used to examine the numbers of viable and dead cells in each treatment. KB cells (1×10^5^ cells/well) cultured in 6-well plates were treated with various concentrations of capsaicin (50, 100, 150, 200 and 250 μM) or DMSO for 24, 48 and 72 h. After harvesting, cell suspensions were thoroughly mixed with equal volumes of a 0.4% trypan blue solution (GIBCO). Viable and dead cells were counted using a hemacytometer under an inverted phase contrast microscope (Nikon Eclipse T*i*-U; Nikon Instruments, Kanagawa, Japan).

### Detection of apoptotic cells

Cells cultured in 24-well plates were treated with capsaicin (150 and 250 μM) or DMSO for 24 and 48 h. Following fixation in freshly prepared ice-cold 4% paraformaldehyde (Sigma) and washing with PBS, 1 μg/ml Hoechst 33342 (Cell Signaling Biotechnology Inc., Beverly, MA, USA) was used to stain the cells. Images were acquired under an inverted fluorescence microscope equipped with a digital camera (Nikon Eclipse T*i*-U; Nikon Instruments). The percentage of apoptotic cells were scored by counting at least 200 cells for each treatment.

### Cell cycle analysis

To detect the proportion of KB cells in different phases of the cell cycle, the DNA content in KB cells was detected by propidium iodide staining (Sigma) and flow cytometry. KB cells seeded in 6-well plates were incubated with capsaicin (150 and 250 μM) or DMSO for 24, 48 and 72 h, and then collected, washed with PBS, and fixed with 70% ethanol at −20°C overnight. After centrifugation, the cells were resuspended in PBS containing 4 μg/ml propidium iodide, 0.1 mg/ml RNase A (Amresco Inc., Solon, OH, USA) and 0.1% Triton X-100 (Sigma), and then incubated at room temperature for 1 h in the dark. Finally, the cell cycle distribution of cells from each treatment was determined by flow cytometry (Beckman FC500; Beckman Instruments, Fullerton, CA, USA) with CXP software. The percentage of cells in different phases of the cell cycle was analyzed by MultiCycle DNA Content and Cell Cycle Analysis Software.

### Measurement of mitochondrial membrane potential (ΔΨm)

To elucidate the role mitochondria play in KB cell death after treatment with capsaicin, changes of the mitochondrial membrane potential were detected by flow cytometry using the fluorescent dye 3,3’-dihexyloxacarbocyanine iodide (DiOC_6_) (Sigma). KB cells (3×10^4^ cells/well) were seeded in 12-well plates. After incubation with various concentrations of capsaicin (100, 200 and 250 μM) or DMSO for 24 and 48 h, the cells were washed with PBS and resuspended in 10 nM DiOC_6_. After incubation at 37°C for 30 min, the cells were immediately analyzed by flow cytometry.

### Immunoblotting

After treatment of KB cells with capsaicin (50, 100, 150 and 250 μM) or DMSO for 24 h, the cells were collected by trypsinization, washed twice with cold PBS, and then lysed with ice-cold lysis buffer consisting of 50 mM Tris–HCl (pH 7.4), 0.1% SDS, 150 mM NaCl, 1 mM EDTA (Merck), 1% Triton X-100 and a protease inhibitor cocktail (BioVision Research Products, Mountain View, CA, USA) of 0.001% (w/v) aprotinin, 0.001% (w/v) leupeptin, 0.00035% (w/v) pepstain A, and 0.085% (w/v) PMSF. Cell lysates were kept on ice for 30 min after gentle vortexing, and then centrifuged at 11,752 g for 10 min at 4°C. The supernatants were immediately analyzed by western blotting or stored at −80°C until use. The protein concentration was measured by a Bio-Rad assay according to the manufacturer’s instructions. For western blot analysis, equal amounts of proteins (20~50 μg) from each treatment were loaded on 12% SDS-PAGE gels. SDS-PAGE and electrophoretic transfer of proteins onto PVDF membranes (Millipore, Billerica, MA, USA) were performed with Hoefer and Bio-Rad apparati, respectively. PVDF membranes were soaked for 1 h in blocking buffer consisting of 5% non-fat milk powder or 5% bovine serum albumin (Sigma) in 1× PBS containing 0.5% Tween-20. Then, PVDF membranes were incubated with antibodies against caspase 8 (Santa Cruz Biotechnology Inc., Santa Cruz, CA, USA), caspase 9, caspase 3, PARP (Cell Signaling), and β-actin (Sigma) for 1 h or overnight. Membranes were then incubated with the appropriate secondary antibody conjugated to horseradish peroxidase for 1 h. After the membranes were exposed to reagents for ECL immunodetection (Millipore), the labeled proteins were detected by autoradiographic film (Eastman Kodak Co., Rochester, NY, USA).

### Statistical analysis

The Student’s *t*-test was performed to determine the statistical significance of the difference between capsaicin- and DMSO-treated groups. Results were represented as the mean ± SD and considered significant at P < 0.05, unless stated otherwise.

## Results

### Capsaicin suppresses growth and induces the death of human KB cancer cells

**Figure 1:**
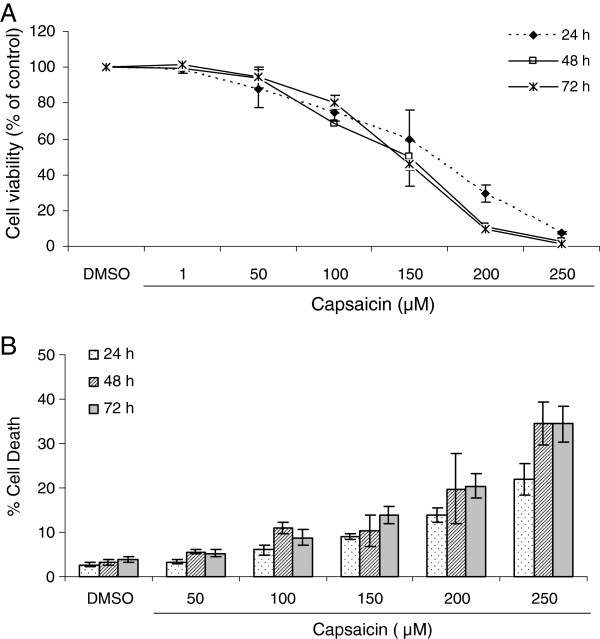
**Capsaicin suppresses cell proliferation/viability and induces cell death in a dose-dependent manner.** (**A**) The effect of capsaicin on the proliferation/viability of KB cells was determined by a SRB assay as described in the Methods. Data are representative of three independent experiments and are expressed as the means ± SE of three replicates. (**B**) The cytotoxic effect of capsaicin on KB cells was determined by trypan blue exclusion as described in the Methods. Data are presented as the means ± SE from three independent experiments.

To determine the effect of capsaicin on the proliferation of KB cells, a preliminary screening was performed to evaluate the proliferation and viability of KB cells using the SRB assay. As shown in Figure 1A, treatment with capsaicin (1, 50, 100, 150, 200 and 250 μM) for 24, 48 and 72 h significantly inhibited the proliferation/viability of KB cells in a dose-dependent manner. In further experiments, we used trypan blue exclusion to evaluate the cytotoxicity of KB cells treated with capsaicin. As shown in Figure 1B, 24, 48 and 72 h exposure to varying concentrations of capsaicin (50, 100, 150, 200 and 250 μM) resulted in a significant dose-dependent reduction of viable cells, indicating that capsaicin exerts a cytotoxic effect on KB cells. Collectively, these data suggest that capsaicin treatment results in dose-dependent growth inhibition and induces cell death in KB cells.

### Capsaicin induces apoptosis in KB cells

**Figure 2:**
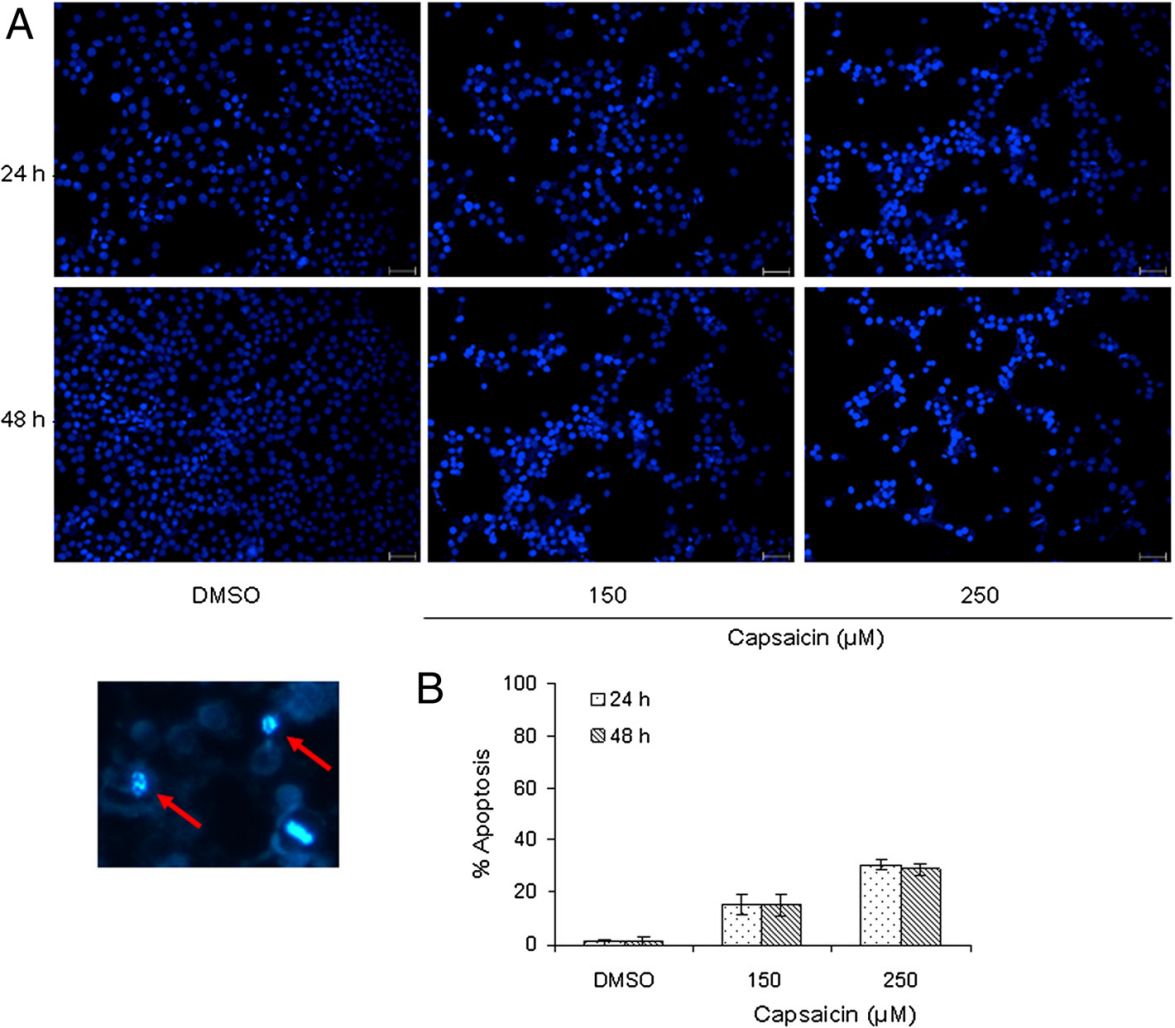
**Capsaicin treatment results in apoptosis of KB cells.** Apoptosis was assessed by Hoechst staining as described in the Methods. (**A**) The morphology of KB cells exposed to capsaicin was observed under a fluorescence microscope. Red arrows indicate apoptotic bodies. (**B**) The number of apoptotic cells was counted among at least 200 cells, and the percentage of apoptotic cells was charted. Data are representative of three independent experiments and are presented as the means ± SE of three independent experiments. Scale bar, 100 μm.

To elucidate whether the capsaicin-induced decrease of proliferation and viability of KB cells was associated with induction of apoptosis, the number of apoptotic cells was assessed by Hoechst staining. Based on the above results, we chose treatment with 150 and 250 μM capsaicin for detection of apoptosis in KB cells. After KB cells were treated with capsaicin for 24 and 48 h, the morphological changes of apoptotic cells, as described previously in [31], were visualized and recorded (Figure 2A). The percentage of apoptotic cells was scored and is summarized in Figure 2B. Similar to the above results, the number of apoptotic cells appeared to show a dose-dependent increase in response to capsaicin treatment. This observation indicates that the anti-proliferative effect of capsaicin on KB cells may be associated with induction of apoptosis.

### Capsaicin induces cell cycle arrest at G2/M phase in KB cells

**Figure 3:**
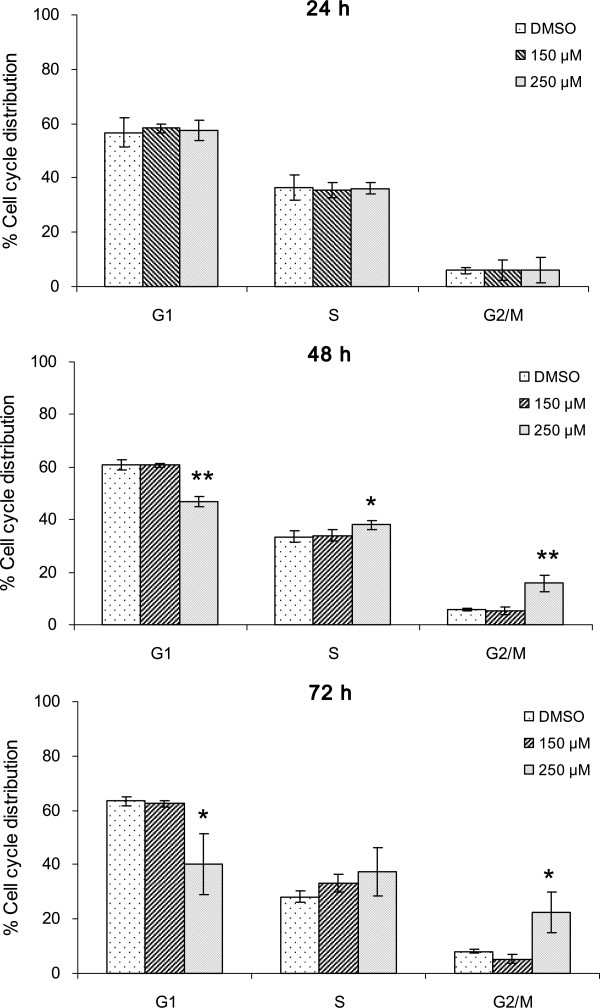
**Capsaicin induces cell cycle arrest at G**_**2**_**/M phase in KB cells.** The cell cycle distribution was determined by propidium iodide staining and flow cytometry as described in the Methods. Data are summarized and presented as the means ± SE of two independent experiments. * P < 0.05, ** P < 0.01.

Cell proliferation is well correlated to the regulation of cell cycle progression. According to the preliminary assays, in which we observed a growth inhibition effect of capsaicin on KB cells (Figure 1), we chose 150 and 250 μM capsaicin to investigate the possible effect of capsaicin on cell cycle progression. Cells harvested at various time points (24, 48 and 72 h) were analyzed by flow cytometry. As shown in Figure 3, there was no significant dose-dependent alteration of cell numbers in different cell cycle phases at 24 h compared with that in the control. This observation suggested that capsaicin did not modulate the cell cycle distribution within 24 h. Capsaicin treatment at 250 μM for 48 and 72 h induced an elevation of the number of cells in the G_2_/M phase of the cell cycle, while the number of cells in the G_1_ phase decreased compared with that in the control. These data indicate that capsaicin treatment leads to the induction of G_2_/M arrest in KB cells.

### Capsaicin induces dissipation of the mitochondrial membrane potential (ΔΨm)

**Figure 4:**
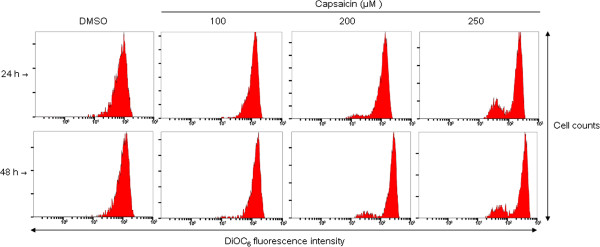
**Capsaicin induces disruption of the mitochondrial membrane potential in KB cells.** Changes of the mitochondrial membrane potential were analyzed by flow cytometry using the fluorescent lipophilic dye DiOC_6_ as described in the Methods. Histograms are representative of two independent experiments.

Mitochondria-initiated events are responsible for the intrinsic pathway of apoptosis. Various stress signals are capable of triggering mitochondrial membrane permeabilization that subsequently leads to the release of cytochrome c and other apoptogenic proteins from the mitochondria to the cytosol. Therefore, to determine whether a mitochondrial response was involved in the capsaicin-induced apoptotic pathway of KB cells, cells treated with capsaicin (100, 200 and 250 μM) for 24 and 48 h were labeled with DiOC_6_ to examine the changes of mitochondrial membrane potential (ΔΨm) by flow cytometry. As shown in Figure 4, peak shifts were observed in a dose-dependent manner in capsaicin-treated cells at 24 and 48 h compared with that in the control, thus confirming depolarization of the mitochondrial membrane potential in KB cells treated with capsaicin.

### Capsaicin induces activation of caspases and PARP

**Figure 5:**
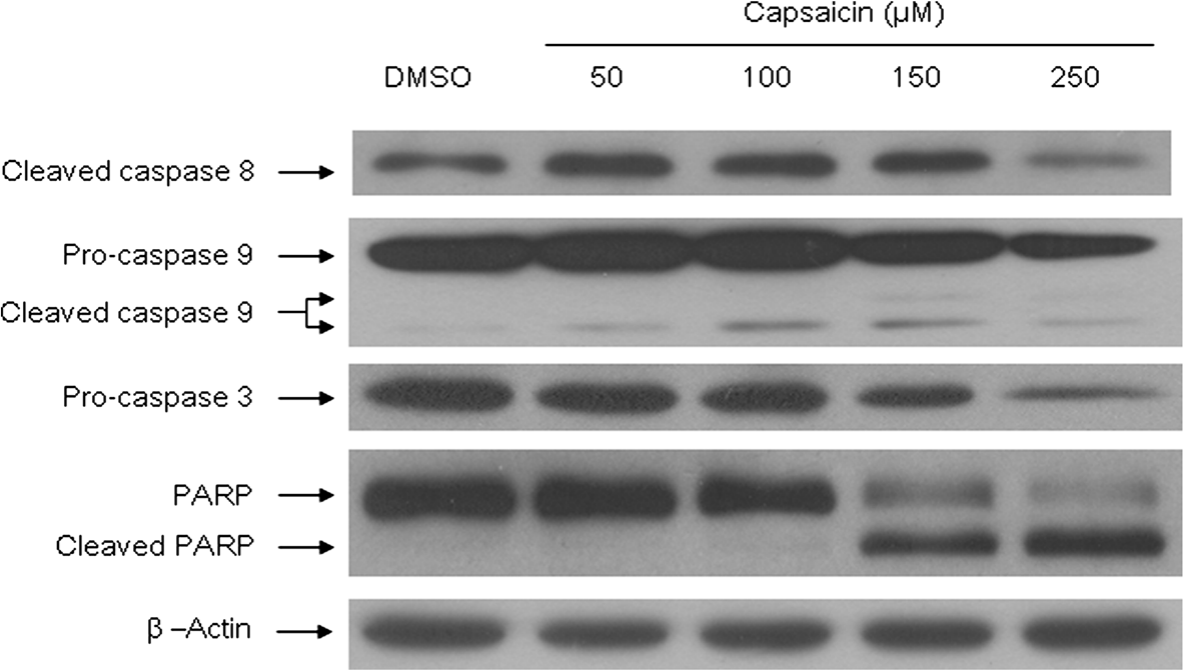
**Apoptosis of KB cells is mediated through the activation of caspases.** The effect of capsaicin on the activation of caspases and PARP was analyzed by immunoblotting as described in the Methods. Equal amounts of each sample were loaded on SDS-PAGE gels. The expression of caspase 3, 8 and 9, PARP, and β-actin were detected using specific antibodies. Equal protein loading was confirmed by stripping and reprobing the blot to detect β-actin expression. The results are representative of at least three independent experiments.

Caspase family members, including caspase 3, 8 and 9, as well as downstream substrates such as PARP, are crucial mediators of the apoptotic process. The previous data indicated a significant decrease in the viability of KB cells at 24 h of exposure to capsaicin. We selected this time point to determine whether induction of apoptosis in KB cells treated with capsaicin was associated with the activation of caspases and PARP. As shown by western blotting in Figure 5, after KB cells were treated with capsaicin for 24 h (50, 100, 150 and 250 μM), capsaicin induced the activation of caspase 9 in the apoptotic intrinsic pathway, resulting in a minor alteration of cleaved caspase 8 in the apoptotic extrinsic pathway compared with that in the control. Moreover, the expression of procaspase 3 decreased and PARP underwent cleavage, thereby confirming apoptosis. The expression of these proteins at the highest dose of capsaicin (250 μM) decreased, except for PARP, which was probably because of extensive cell death or the terminal stage of apoptosis. These data suggest that capsaicin-induced apoptosis in KB cells is associated with caspase 3 and 9.

## Discussion

Capsaicin is a naturally occurring plant phytochemical present in chili peppers. Whether it acts as a carcinogen, co-carcinogen, or anti-carcinogen is controversial [34,35]. Nonetheless, accumulating findings indicate that capsaicin possesses anti-cancer properties in various cancer cell lines [8-29]. The goal of this study was to explore the mechanism underlying capsaicin-induced cell death in human KB cancer cells. Here, we report that treatment of KB cells with capsaicin inhibits their proliferation/viability, halts cell cycle progression, and induces apoptosis through mitochondrial membrane permeabilization and caspase activation.

In the preliminary study, we found that capsaicin treatment markedly reduced the proliferation and viability of KB cells (Figure 1), thus raising the possibility that capsaicin might be a potential chemopreventive or therapeutic agent. Several naturally occurring phytochemicals have been reported to suppress the growth of cancer cells through disruption of cell cycle progression [1,2]. Evading cell cycle arrest is the most frequently observed phenomenon in tumor development. The cell cycle includes a number of checkpoints that act as surveillance mechanisms. Upon cellular stress or DNA damage, these mechanisms induce cells to undergo either cell cycle arrest, activation of repair systems, or apoptotic induction. Checkpoints at G_1_/S and G_2_/M transitions are essential regulatory gates during cell cycle progression [36,37]. Numerous studies have shown a common feature in which exposure to capsaicin induces cell cycle arrest at the G_1_ phase in a variety of human cancer cell lines [8-12,15-17,22,24,25,27,28]. Interestingly, we found that G_2_/M phase arrest of KB cells was observed at the highest capsaicin concentration until 48 h, and no earlier cell cycle arrest was found (Figure 3). This finding suggests that the mode of capsaicin-induced cell cycle arrest depends on the cell type. Presently, the molecular mechanisms of capsaicin-induced cell cycle arrest in KB cells are unknown and require further investigation.

Apart from cell cycle arrest, induction of apoptosis is viewed as a mechanism through which a variety of naturally occurring phytochemicals inhibit tumor growth [1,2]. Recent studies have shown that capsaicin leads to induction of apoptosis in a variety of cancer cell lines [8-29]. The data obtained in our study demonstrated dose-dependent induction of apoptosis in KB cells treated with capsaicin. We found that capsaicin triggered the activation of apoptosis prior to G_2_/M phase arrest in KB cells (Figures 2, 3 and 5). As mentioned above, control of cell cycle progression is a strategy to halt tumor growth [1,2,36,37]. However, based on our data (Figures 1 and 3), it appears that inhibition of uncontrolled cell cycle progression plays a limited role in the inhibitory effect of capsaicin on KB cell growth. In fact, suppression of KB cell growth by capsaicin may be more correlated with induction of apoptosis.

Mitochondria-initiated responses are involved in regulation of the intrinsic apoptotic pathway [31,32]. Furthermore, the metabolic activities of mitochondria in cancer cells are distinct from those in normal cells, and are considered to be a biologically significant source of apoptotic failure that is closely associated with chemo- and radio-resistances in cancer therapy. Therefore, induced dysfunction of mitochondria is an attractive strategy for the control of cell proliferation. The loss of mitochondrial membrane stabilization is a key event to eliminate cancer cells [38,39]. Although exposure to capsaicin shows a slight change of mitochondrial membrane potential in MCF-7 breast cancer cells [26], multiple studies have revealed that capsaicin treatment leads to disruption of the mitochondrial membrane potential in various cancer cell lines [9,10,12,14-17,19,20,22]. These studies are consistent with our observation in which KB cells treated with capsaicin underwent loss of the mitochondrial membrane potential (Figure 4). Once the mitochondrial outer membrane is permeable, proapoptotic proteins such as cytochrome c are released into the cytosol from the intermembrane space, resulting in activation of caspase 9. Western blot analysis indicated the involvement of caspase 9 in capsaicin-induced apoptosis of KB cells (Figure 5). The other route of apoptosis, the extrinsic pathway, is initiated by the binding of ligands to death receptors. The transmembrane death signal induces formation of the DISC through adaptor proteins and subsequently leads to activation of caspase 8. Our data showed that caspase 8 did not play a role in apoptosis of KB cells treated with capsaicin (Figure 5). Both intrinsic and extrinsic pathways converge on common factors including caspase 3. Upon activation of caspases 3, substrates such as PARP are cleaved, ultimately leading to apoptotic cell death [31,32]. Our results revealed that caspase 3 may be associated with capsaicin-induced apoptosis of KB cells (Figure 5). Collectively, mitochondria and caspase members are feasible targets of capsaicin for apoptosis of human cancer cells.

## Conclusions

Our study indicates that exposure of human KB cancer cells to capsaicin reduces cell viability, induces cell cycle arrest at G_2_/M phase, and activates apoptosis that involves mitochondria and caspase members. However, capsaicin-induced cell cycle arrest may not be effective for inhibition of KB cell growth. The apoptosis of KB cells treated with capsaicin is associated with the induction of caspase 3 and 9, as well as dissipation of the mitochondrial membrane potential. In summary, these findings suggest that capsaicin possesses an anti-cancer activity and may be a potential candidate as an anti-cancer agent.

## Competing interests

The authors have no competing interests.

## Authors’ contributions

CHL, WCL, and MKC conceived and designed the study. WCL and YCC performed the experiments. CHL, WCL, and CWW analyzed the data. CHL and WCL drafted the manuscript. CWW revised the manuscript. MKC provided comments and editorial review of the manuscript. All authors read and approved the final manuscript.

## Pre-publication history

The pre-publication history for this paper can be accessed here:

http://www.biomedcentral.com/1472-6882/13/46/prepub
